# The role of HIF-1α/HO-1 pathway in hippocampal neuronal ferroptosis in epilepsy

**DOI:** 10.1016/j.isci.2023.108098

**Published:** 2023-09-29

**Authors:** Zhen Liang, Zhaoshi Zheng, Qi Guo, Meng Tian, Jing Yang, Xiu Liu, Xiaojuan Zhu, Songyan Liu

**Affiliations:** 1 Department of Neurology, China-Japan Union Hospital of Jilin University, Changchun, China; 2 Key Laboratory of Molecular Epigenetics, Ministry of Education and Institute of Cytology and Genetics, Northeast Normal University, Changchun, China

**Keywords:** Health sciences, Molecular biology, Neuroscience, Cell biology, Metabolomics

## Abstract

Epilepsy, a common central nervous system disorder, remains an enigma in pathogenesis. Emerging consensus designates hippocampal neuronal injury as a cornerstone for epileptogenic foci, pivotal in epileptic genesis and progression. Ferroptosis, a regulated cell death modality hinging on iron, catalyzes lipid reactive oxygen species formation through iron and membrane polyunsaturated fatty acid interplay, culminating in oxidative cell death. This research investigates the role of hypoxia-inducible factor (HIF)-1α/heme oxygenase (HO)-1 in hippocampal neuron ferroptosis during epilepsy. Untargeted metabolomics exposes metabolite discrepancies between epilepsy patients and healthy individuals, unveiling escalated oxidative stress, heightened bilirubin, and augmented iron metabolism in epileptic blood. Enrichment analyses unveil active HIF-1 pathway in epileptic pathogenesis, reinforced by HIF-1α signaling perturbations in DisGeNET database. PTZ-kindled mice model confirms increased ferroptotic markers, oxidative stress, HIF-1α, and HO-1 in epilepsy. Study implicates HIF-1α/HO-1 potentially regulates hippocampal neuronal ferroptosis, iron metabolism, and oxidative stress, thereby promoting the propagation of epilepsy.

## Highlights

• Combined omics analysis and animal experiments
• Omics analysis indicated occurrence of ferroptosis in epilepsy patients
• Animal experiments confirmed the hypothesis of omics analysis
• The key role of HIF-1α/HO-1 pathway in epilepsy

## Graphical abstract

**Figure:**
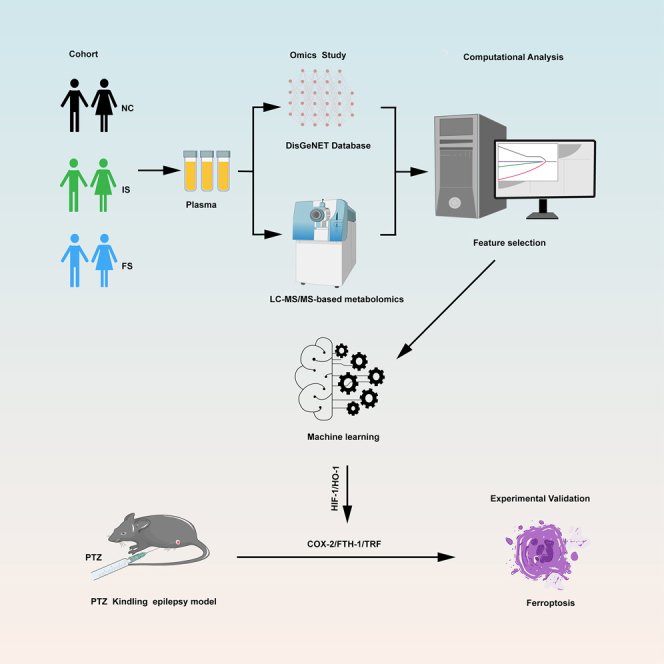

## Introduction

Epilepsy is a common chronic disease of the central nervous system (CNS) characterized by repeated seizures caused by abnormal electrical activity in the brain. Despite long-term efforts in the study of epilepsy, the exact pathogenic mechanism is still not fully elucidated.^1^^,^^2^ Epileptic seizures can cause neuronal damage, ion pathway dysfunction, mossy fiber budding, gliosis, synaptic plasticity, and inflammatory response, especially hippocampal neuron damage. In turn, hippocampal neuron damage can further promote seizures. Therefore, signaling pathways involved in hippocampal neuron damage are considered potential targets for alleviating seizures or delaying the development of epilepsy.^3^ Iron is an important trace element that is essential for maintaining neuronal function and metabolic processes in the CNS.^4^ However, when iron, especially ferrous iron, accumulates excessively in the CNS, it can lead to oxidative stress and cell death. Recent studies have shown that iron accumulation is strongly associated with the occurrence and development of a variety of neurological disorders, including epilepsy.^5^

Metabolomics, which focuses on small molecule metabolites (<1000 Da) present in biological systems, is a powerful tool used to comprehensively evaluate the dynamic changes of metabolites after endogenous or exogenous interference. Metabolomics analysis can be used to understand the metabolic characteristics of an organism in physiological or pathological states, as well as the interaction and regulatory network between metabolites.^6^ Metabolomics can provide comprehensive metabolic information and provide important support for understanding the physiological status, disease mechanism and drug response of organisms, and is widely used in medicine, medicine and agriculture.^7^^,^^8^ However, metabolomics approaches still have limitations. They only focus on the terminal variation caused by interferences, but ignore the endogenous mechanisms of metabolite activity.^9^ DisGeNET database is a comprehensive gene-disease association (GDA) database that provides extensive information on variants and genes associated with human disease. Recently, DisGeNET database has been widely applied to investigate the potential target genes of various diseases.^10^ Therefore, the integration of metabolomics and DisGeNET database can help overcome the limitations due to the lack of experimental basis for the former and the lack of molecular mechanism explanations for the latter, and will contribute to a better understanding of the pathogenesis of epilepsy. In this study, an untargeted metabolomics approach was employed to comprehensively and unbiasedly detect differential metabolites in the peripheral blood of epilepsy patients compared to the normal population. The results revealed elevated levels of free fatty acids, decreased levels of glutamine, increased levels of bilirubin, and enhanced iron metabolism in the peripheral blood of epilepsy patients. Further functional enrichment analysis of the differential metabolites highlighted the significant activation of hypoxia-inducible factor (HIF) -1 pathway in the pathological state of epilepsy. Additionally, we also used the DisGeNET database to collect target genes related to human epilepsy for functional enrichment analysis, and the analysis results further support that HIF-1 is an important disease target for human epilepsy.

HIF-1 is a crucial transcription factor involved in regulating cellular adaptation to low oxygen environments. Comprising two subunits, HIF-1α and HIF-1β, HIF-1α serves as the primary functional subunit.^11^ HIF-1α orchestrates the transcriptional activation of over 60 genes, including heme oxygenase (HO)-1.^12^ HO-1 is a stress-inducing enzyme that metabolizes heme into biliverdin (rapidly converted to bilirubin), carbon monoxide, and ferrous iron (which induces an increase in ferritin).^12^ Recent research has highlighted the significant role of HIF-1α/HO-1-mediated ferroptosis in mouse diabetic nephropathy^13^ and infertility.^14^ However, the interaction between HIF-1α and HO-1, along with their neurotoxic mechanisms associated with hippocampal neuronal ferroptosis in epilepsy, remains unclear. Therefore, we propose a scientific hypothesis that the HIF-1α/HO-1 pathway promotes the occurrence and development of epilepsy by mediating hippocampal neuronal ferroptosis.

This study combines metabolomics with human disease public databases to uncover the potential role of the HIF-1α/HO-1 pathway in hippocampal neuronal ferroptosis during epilepsy. Subsequently, we will employ various experimental approaches *in vivo*, including molecular biology, cell biology, and behavioral studies, to validate the findings from the metabolomics research and further elucidate the critical role of the HIF-1α/HO-1 pathway in the pathogenesis of hippocampal neuronal ferroptosis-related epilepsy. This research holds promise for enhancing our understanding of the mechanisms underlying epilepsy and providing a theoretical basis for future development of novel therapeutic strategies and drugs.

## Results

### Sample characteristic

The study consisted of a total of eighty-two subjects: sixty-two epilepsy patients categorized into infrequent seizure (IS) (n = 31) and frequent seizures (FS) (n = 31) based on the frequency of their seizures. Additionally, 20 healthy volunteers served as NC (n = 20). There were no significant differences in age and sex among the three groups. There were no statistically significant differences in age of first onset or seizure type between the two groups. (Table 1).

**Table 1:** Demographic and clinical characteristics of enrolled subjects

Demographic and clinical characteristics	NC （n = 20）	IS （n = 31）	FS （n = 31）	p-value
Age (mean ± SD)	28.05 ± 4.17	31.39 ± 13.77	35.10 ± 18.06	0.218^a^
Male/Female Ratio	10/10	18/13	18/13	0.819^b^
Age at onset (mean ± SD)		24.55 ± 16.13	25.97 ± 16.84	0.736^c^
Seizure type (n)				
Focal		11	6	0.376^b^
Generalized		14	18	0.376^b^
Mixed		6	6	0.376^b^

NC, Normal control; IS, Infrequent seizure; FS, Frequent seizures.

n, number of samples; SD, Standard deviation.

a p value calculated using ANOVA test.

b p value calculated using chi-square test.

c p value calculated using T-test.

### Untargeted metabolomic differential metabolite analysis

**Figure 1:**
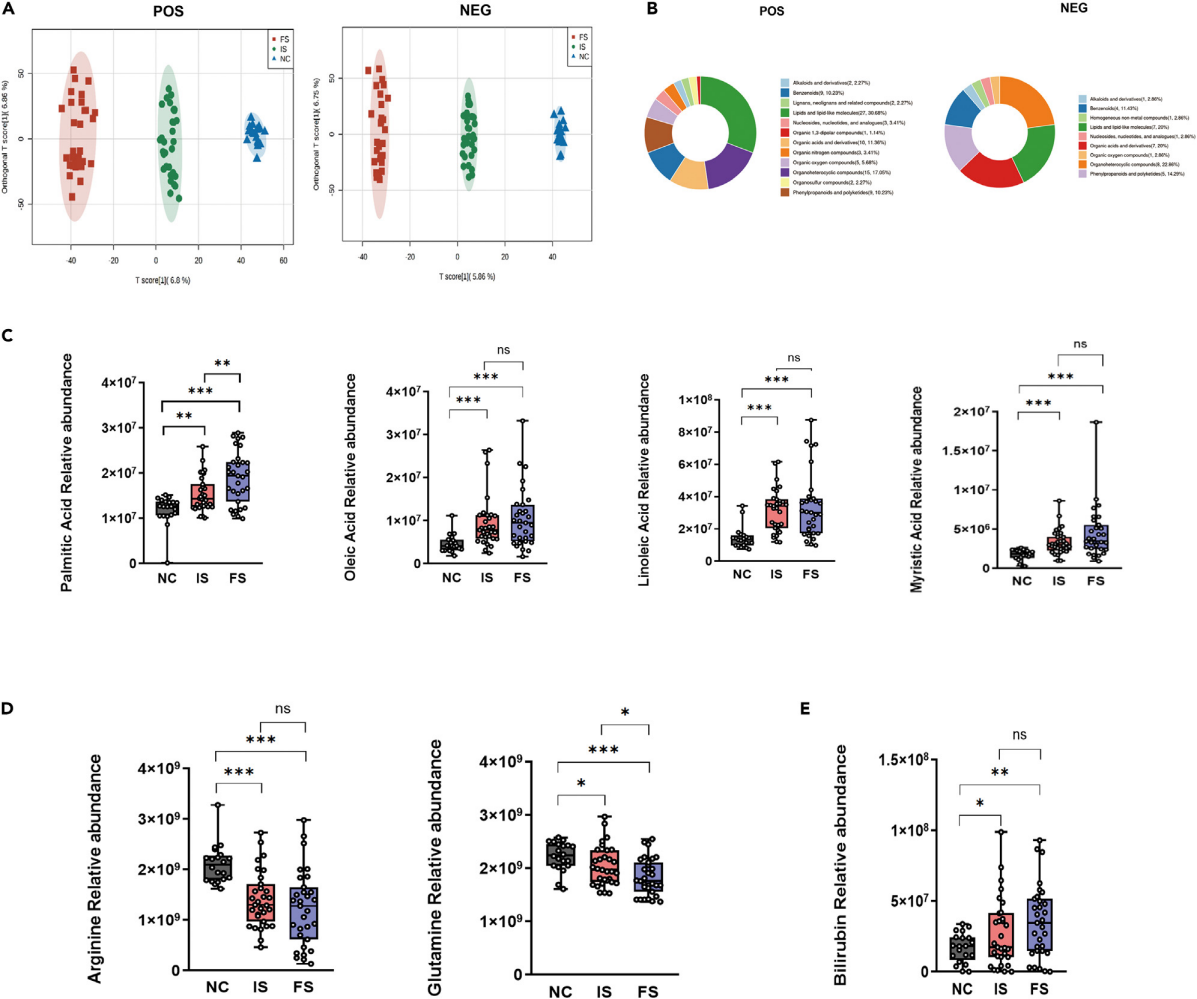
Untargeted metabolomic differential metabolites analysis among NC, IS, and FS groups (A) OPLS-DA score in positive and negative ion mode. (B) HMDB classification ring maps in positive and negative ion mode. (C‒E) Relative abundance of several representative differential metabolites. One-way ANOVA was used to compare three groups, followed by conducting pairwise comparisons between groups using Welch’s T-test. The data are presented as mean ± SEM. ∗p < 0.05, ∗∗p < 0.01, ∗∗∗p < 0.001, and ns for no significance.

Firstly, we constructed an OPLS-DA model based on the untargeted metabolomics results of epilepsy patients and control subjects (Figure 1A). The results revealed significant inter-group differences and good intra-group reproducibility in the differential metabolites. The differentially expressed metabolites, identified based on the criteria of VIP score > 1.0 and p < 0.05, have the potential to serve as biomarkers for diagnosing epilepsy. A total of 153 significantly altered metabolites were identified in the positive ion mode, and 76 differential metabolites were identified in the negative ion mode when comparing the NC, IS, and FS group (Table S1). We can find from the HMDB classification ring maps that the differential metabolites of epilepsy patients are mostly concentrated in lipids and small lipid molecules (30.68%), organic heterocyclic compounds (17.05%), organic acids and derivatives (11.36%) in positive ion mode. While in the negative ion mode, differential metabolites were mainly concentrated in organic heterocyclic compounds (22.86%), organic acids and derivatives (20%), lipids and small lipid molecules (20%) (Figure 1B). In terms of fatty acids metabolism, compared to the NC group, the epilepsy groups showed elevated levels of palmitic acid, oleic acid, linoleic acid, and myristic acid. Furthermore, the levels of these metabolites increased with the frequency of epileptic seizures. It is worth noting that the difference in palmitic acid levels between the FS group and the IS group was statistically significant (p < 0.05) (Figure 1C). Regarding amino acid metabolism, glutamine and arginine levels in peripheral blood of epileptic patients were lower than those of NC group. As the frequency of seizures increased, glutamine and arginine levels further decreased. Similarly, there was a statistically significant difference in glutamine levels between the FS and IS groups (p < 0.05) (Figure 1D). Additionally, the epilepsy groups showed elevated levels of bilirubin compared to the control group. Moreover, as the frequency of epileptic seizures increased, there was a trend of further elevation in bilirubin levels (Figure 1E). HO-1 can metabolize heme into bilirubin, carbon monoxide, and ferrous iron. Heme iron represents the main source of iron in the body, accounting for approximately 67% of total body iron.^15^ Enhanced heme metabolism can lead to changes in iron content in the body. Therefore, the alterations in peripheral blood metabolites in epilepsy patients suggest the presence of abnormal iron metabolism and oxidative stress, indicating a potential role of ferroptosis in the development of epilepsy.

### KEGG pathway enrichment analysis of differential metabolites

**Figure 2:**
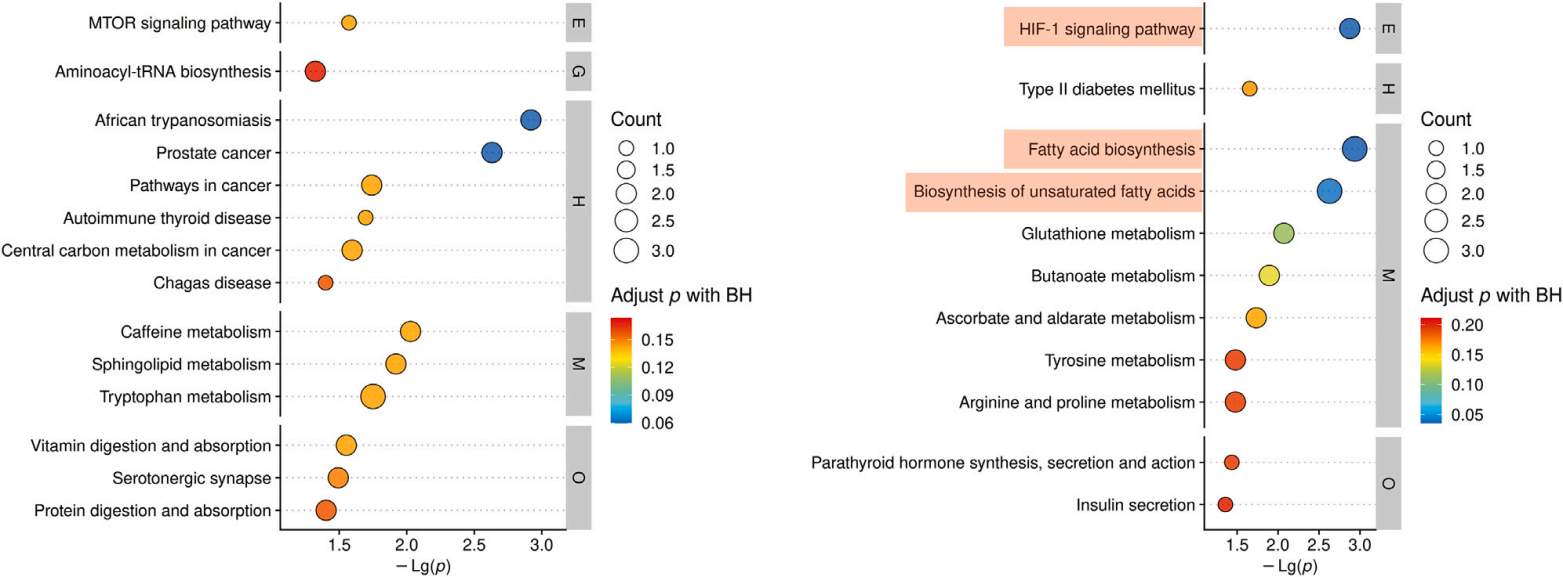
KEGG pathway enrichment analysis of differential metabolites in positive and negative ion mode

We performed Kyoto encyclopedia of genes and genomes (KEGG) pathway enrichment analysis on the differential metabolites. In the positive ion mode, no significantly enriched pathways were identified among the differential metabolites. However, in the negative ion mode, the HIF-1 signaling pathway, fatty acid biosynthesis signaling pathway, and unsaturated fatty acid biosynthesis signaling pathway were significantly enriched (Adjusted p < 0.05) (Figure 2). This is consistent with previous knowledge, as ferroptosis involves excessive reactive oxygen species (ROS) production and accumulation through iron-catalyzed lipid peroxidation, leading to oxidative cell death. Furthermore, previous studies have indicated that the increase in unsaturated fatty acids or free fatty acids provides substrates for iron-mediated cell death. Therefore, our functional enrichment results align with existing understanding in the field.

### Screening and functional enrichment analysis of epilepsy-associated genes based on DisGeNET database

**Figure 3:**
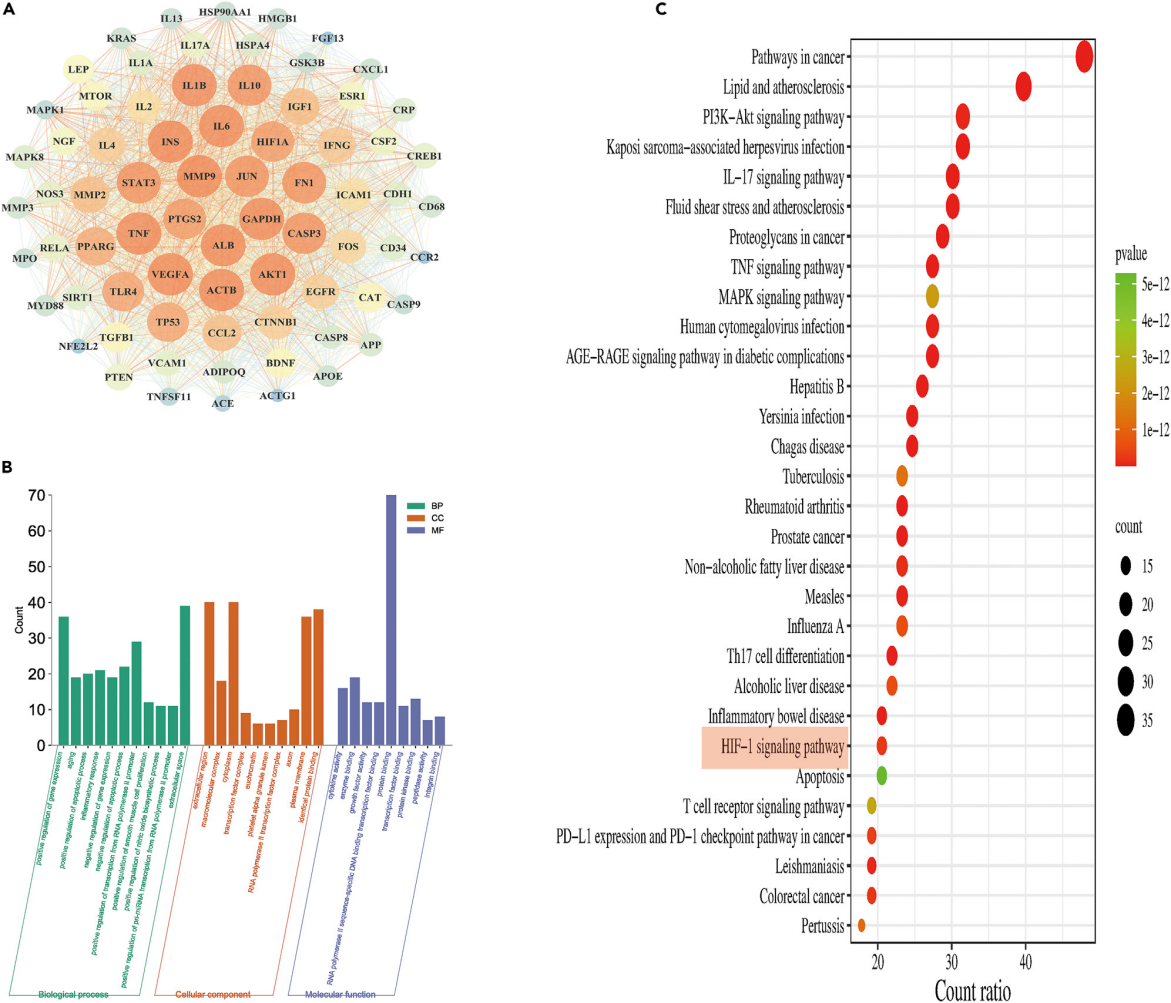
Screening and functional enrichment analysis of epilepsy-associated genes based on DisGeNET database (A) PPI network for epilepsy-associated genes. (B) GO analysis. (C) KEGG analysis.

DisGeNET database is a comprehensive GDA database that provides a wealth of information on variations and genes associated with human diseases. Using “epilepsy” as a keyword, we collected 1215 disease target genes related to epilepsy from the DisGeNET database (Table S2). Additionally, we constructed a protein-protein interaction (PPI) network using the String database and visualized the PPI network using Cytoscape 3.7.2 software. By applying selection criteria (degree cutoff = 2, node score cutoff = 0.2, K-core = 2, Max. Depth = 100), we identified a core network and 73 hub target genes (Figure 3A). Next, we conducted functional and pathway enrichment analysis for the 73 hub target genes associated with epilepsy. The Gene Ontology (GO) analysis yielded a total of 540 biological processes (BP) terms, 62 cellular components (CC) terms, and 68 molecular functions (MF) terms (Table S3). The GO enrichment results were visualized as a histogram based on the p value (TOP30). In terms of BP, the predominant themes encompassed positive regulation of gene expression, aging, positive regulation of apoptosis, inflammatory response, and NO synthesis. CC analysis primarily highlighted extracellular regions, macromolecular complexes, cytoplasm, plasma membrane, and transcription factor complexes. MF analysis mainly focused on cytokine binding, enzyme binding, transcription factor binding, protein binding, and protein kinase binding (Figure 3B). Furthermore, the KEGG analysis identified 153 significantly enriched signaling pathways (Table S4). Based on the p value (TOP30), we visualized The KEGG enrichment results using a bubble plot. Notably, the HIF-1 signaling pathway also exhibited significant enrichment (p < 0.0001) (Figure 3C). Key molecules enriched in the HIF-1 pathway include HIF-1α, NOS3, STAT3, IGF1, EGFR, MTOR, RELA, INS, VEGFA, IL6, IFNG, AKT1, MAPK1, GAPDH, and TLR4. These findings further support the notion that the HIF-1α signaling pathway may play a crucial role in ferroptosis and the development of epilepsy.

### Validation of hippocampal neurons ferroptosis in PTZ kindling epilepsy mice models

**Figure 4:**
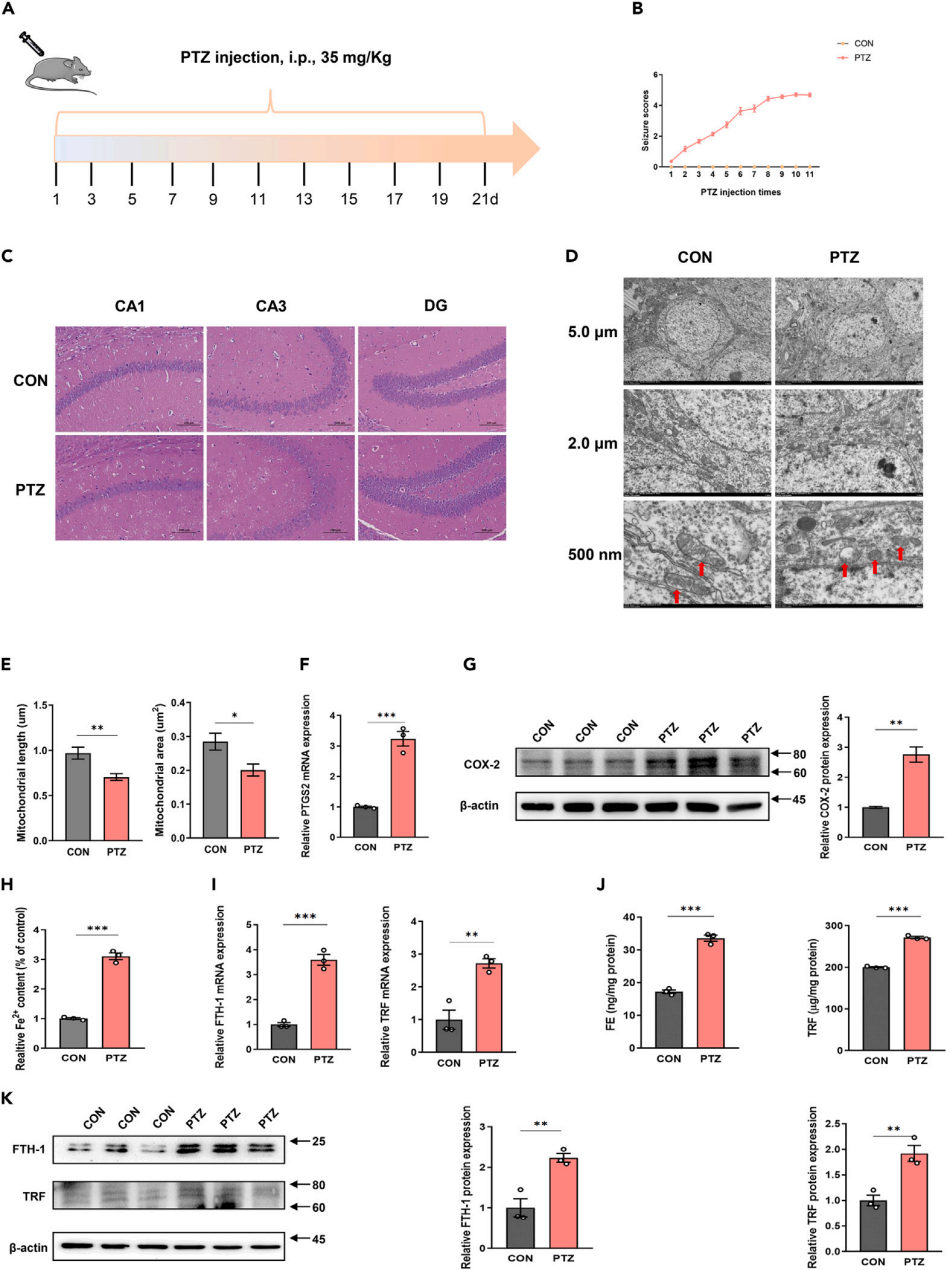
Validation of hippocampal neurons ferroptosis in PTZ kindling epilepsy mice models (A) Flow schematic depiction of study design. (B) Statistical chart of seizure score changes after each PTZ injection in epileptic model group. (C) HE staining of CA1, CA3, and DG regions in hippocampus of PTZ and CON group. (D) TEM results of hippocampus in PTZ and CON group with 5.0 μm, 2.0 μm, and 500 nm scale. (E) The quantification of mitochondrial length and area in hippocampus. (F and G) RT-qPCR and western blot results for PTGS2/COX-2 in hippocampus. (H) Relative Fe2+ accumulation in hippocampus. (I‒K) RT-qPCR, ELISA and western blot results for FTH-1 and TRF in hippocampus. Unpaired two-tailed Student’s t-test was used to compare two groups. The data are presented as mean ± SEM. ∗p < 0.05, ∗∗p < 0.01, and ∗∗∗p < 0.001.

We successfully established the PTZ kindling epilepsy mice models according to the previous stable model establishment method. The flow schematic depiction of study design is showed in Figure 4A. Compared with CON group, the epileptic seizure score of mice in the PTZ group was significantly higher, in addition, the incidence and duration of epilepsy of mice in the PTZ group met the criteria for successful modeling (Figure 4B, Table 2). We observed the structure of CA1, CA3, and DG regions in the hippocampus of PTZ and CON group by HE staining. In the control group, the neurons in CA1, CA3, and DG regions of the hippocampus were arranged neatly, the structure was complete, the chromatin distribution was uniform, the nucleolus was clear, and no obvious neuron loss was observed. However, in the PTZ group, the neurons in CA1 and CA3 regions of the hippocampus were disordered, some neurons were missing, the number was reduced, the shape of neurons was abnormal, the regular round and oval gradually shrank and the color was deepened, the nucleus was shrunk, the large nucleolus disappeared, and the neurons were vacuolized. In addition, the damage in DG region was not obvious (Figure 4C). We evaluated the subcellular structure of hippocampal neurons in two groups of mice by TEM. The results showed that the nucleus size of hippocampal neurons in both groups was normal and there was no chromatin condensation. However, compared with the CON group, the mitochondria of hippocampal neurons in the PTZ group became smaller, the membrane density increased, and the mitochondrial ridge decreased or disappeared, which was consistent with the mitochondrial change characteristic of ferroptosis reported in the literature (Figure 4D). In contrast to the control group, the mice in PTZ group displayed modified mitochondrial structure, showing significant reductions in both mitochondrial length and area (Figure 4E). This suggests an abnormality in mitochondrial dynamics. Then, we detected the expression of ferroptosis marker gene prostaglandin-endoperoxide synthase 2/cyclooxygenase 2 (PTGS2/COX2) in the two groups of mice by RT-qPCR and western blot. We found that at both transcription and translation levels, PTGS2/COX-2 expression were significantly elevated in mice in the PTZ group (Figures 4F and 4G). In addition, Fe^2+^ accumulation in the hippocampal tissues of two groups of mice was also detected by iron content detection kit. We found that compared with the CON group, the relative Fe^2+^ content in the hippocampus of mice in the PTZ group was significantly increased (Figure 4H). As for ferritin (FE), because FE has heavy chain (FTH-1) and light chain (FTL), FTH1 mainly exists in neurons, while FTL is mainly expressed in microglia.^16^ Therefore, we detected the expression levels of FTH-1 and transferrin (TRF) in the hippocampus of two groups of mice. According to the results of RT-qPCR, transcription levels of FTH1 and TRF in the hippocampus of mice in the PTZ group were significantly increased compared with the CON group (Figure 4I). ELISA results showed that the contents of FE and TRF in the hippocampus of mice in the PTZ injection group were significantly increased (Figure 4J). Western blot results also showed that the expressions of FTH-1 and TRF proteins in the hippocampus of mice in the PTZ group were significantly increased (Figure 4K).

**Table 2:** Behavioral statistical results of epileptic seizure in different treatment groups

Group	N	Percentage of kindled (%)	Latency time of kindled (d)	Last seizure scale ≥4	
				Latency time (s)	Duration time (s)
CON	30	0	–	–	–
PTZ	30	83.3	16.48 ± 2.19	208.36 ± 15.78	62.96 ± 6.08

### Validation of excessive oxidative stress of hippocampus in PTZ kindling epilepsy mice models

**Figure 5:**
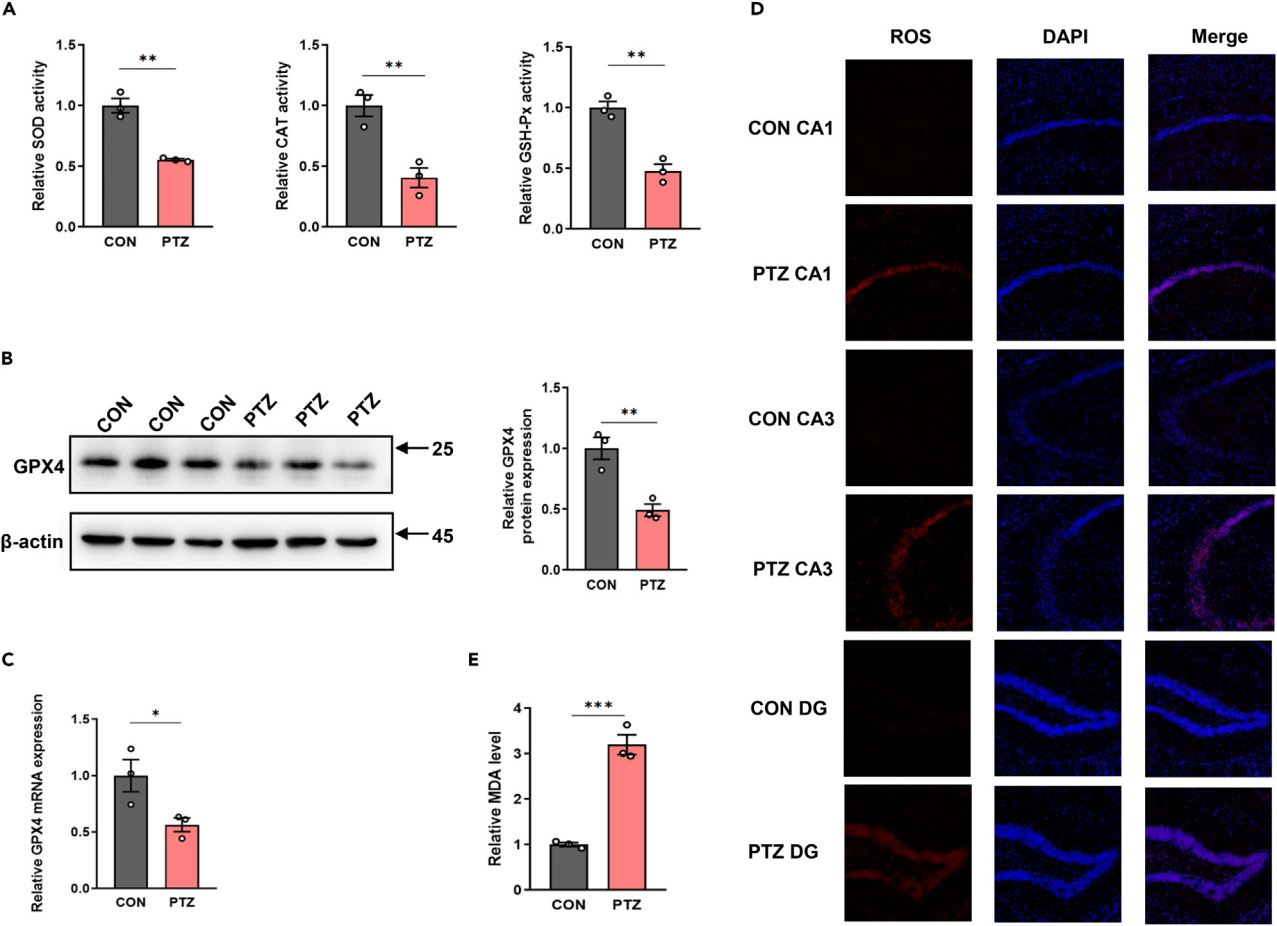
Validation of excessive oxidative stress of hippocampus in PTZ kindling epilepsy mice models (A) Relative activity of antioxidant enzymes in hippocampus, including SOD, CAT, and GSH-Px. (B and C) western blot and RT-qPCR results for GPX4 in hippocampus. (D) ROS staining of CA1, CA3, and DG regions in hippocampus. (E) Relative MDA content hippocampus. Unpaired two-tailed Student’s t-test was used to compare two groups. The data are presented as mean ± SEM. ∗p < 0.05, ∗∗p < 0.01, and ∗∗∗p < 0.001.

Aiming to observe the antioxidant ability of hippocampal tissue after epilepsy, we firstly measured the activities of SOD, CAT, and GSH-Px. Compared with CON group, the activities of these three antioxidant enzymes were significantly decreased in PTZ group (Figure 5A). GPX4, which has a broader substrate preference, is the only enzyme described that can directly reduce complex phospholipid hydroperoxides. Therefore, we measured GPX4 expression levels in hippocampal tissues of two groups of mice from transcriptional and protein levels, respectively. The results showed that GPX4 mRNA and protein expression in hippocampus of mice in PTZ group were significantly lower than those in CON group (Figures 5B and 5C). We also used ROS frozen section staining method to detect ROS content in hippocampal CA1, CA3, and DG regions of mice in CON group and PTZ group, respectively. The results showed that ROS content in hippocampal CA1, CA3, and DG regions of mice in PTZ group was significantly increased compared with the CON group (Figure 5D). MDA is a natural product of lipid oxidation, and the content of MDA reflects the level of lipid oxidation. Therefore, the determination of MDA is widely used as an index to react lipid oxidation. We found that compared with the CON group, MDA content in the hippocampus of mice in the PTZ group was significantly increased (Figure 5E), indicating lipid peroxidation in the hippocampus of mice in the PTZ group. In conclusion, the mice in PTZ group showed typical excessive oxidative stress and ferroptosis in the hippocampus.

### Validation of the activation of HIF-1α/HO-1 pathway in hippocampus in PTZ kindling epilepsy mice models

**Figure 6:**
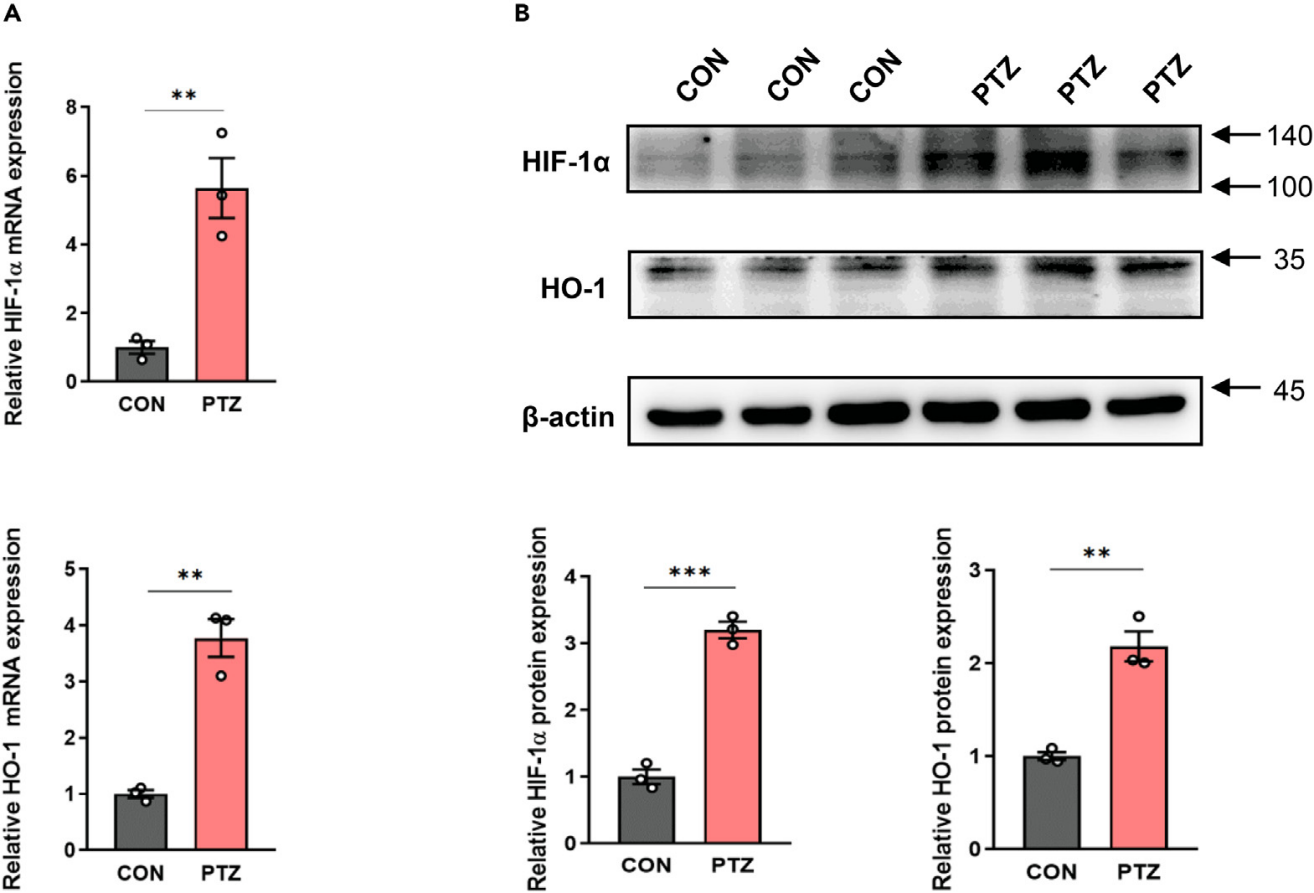
Validation of the activation of HIF-1α/HO-1 pathway in hippocampus in PTZ kindling epilepsy mice models (A and B) RT-qPCR and western blot results for HIF-1α and HO-1 in hippocampus. Unpaired two-tailed Student’s t-test was used to compare two groups. The data are presented as mean ± SEM. ∗p < 0.05, ∗∗p < 0.01, and ∗∗∗p < 0.001.

To verify that HIF-1α/HO-1 pathway is activated in epilepsy, we performed RT-qPCR and western blot assays to detect hippocampal tissues of PTZ and CON mice at transcriptional and translational levels. RT-qPCR results showed the mRNA levels of HIF-1α and HO-1 in the hippocampus of mice in the PTZ group were significantly increased compared with the CON group (Figure 6A). Western blot results also showed that the protein expressions of HIF-1α and HO-1 in the hippocampus of mice in the PTZ group were significantly increased (Figure 6B). These results suggest that HIF-1α/HO-1 pathway is significantly activated in the hippocampus of PTZ kindling epileptic mice.

## Discussion

Epilepsy is a chronic brain disease caused by highly synchronized abnormal discharge of brain neurons, which has the characteristics of recurrence, seizure, transitory, and stereotyped.^17^ As estimated, the current prevalence of epilepsy has achieved 1% in the general population, with 80% of people with epilepsy living in low- and middle-income countries, causing a substantial financial burden.^18^ The pathogenesis of epilepsy remains elusive, with two plausible mechanisms currently being postulated: the ion channel theory and the abnormal network theory. According to the ion channel theory, epilepsy arises from genetic abnormalities that result in dysregulation of excitatory or inhibitory neurotransmitters, leading to aberrant opening of ion channels and subsequent neuronal discharge, culminating in epileptic seizures. While the abnormal network theory proposes that epilepsy originates from neuronal death, wherein the remaining neurons, along with newly generated neurons and proliferating glial cells, form novel networks. When these networks facilitate the formation and propagation of epilepsy, recurrent seizure activity is triggered, perpetuating the cycle of neuronal death and establishing a malignant loop.^19^^,^^20^^,^^21^ Ion channels exert rapid effects and are the primary targets of the antiepileptic drugs commonly used in clinical practice. Although these medications effectively control seizures, their long-term use may lead to adverse reactions such as increased blood drug concentrations, drug resistance, and neurologic and psychiatric impairments. Unfortunately, there is currently no ideal pharmacological intervention that regulates neuronal death to improve epilepsy. Therefore, further exploration of the role and molecular biology mechanisms of neuronal damage in epilepsy holds promise for identifying novel drug targets that could revolutionize the treatment of epilepsy.^18^

Untargeted metabolomics, which involves comprehensive analysis of metabolic products in an organism under specific conditions, holds significant importance in unraveling the pathogenesis of epilepsy and identifying novel therapeutic targets.^22^ Through conducting untargeted metabolomics studies in patients with epilepsy, we can uncover metabolic abnormalities associated with the condition, thereby deepening our understanding of the pathogenesis of epilepsy. In this study, we performed comprehensive metabolomic analysis on peripheral blood samples collected from both epilepsy patients and healthy individuals, aiming to provide insights into the identification of molecular and biological targets for epilepsy treatment. Blood samples are widely used in clinical research on various diseases, including epilepsy. They are often employed as a substitute for brain tissue due to their ease of accessibility. Furthermore, blood samples can effectively reflect the overall physiological changes occurring before and after the onset of the disease.^22^^,^^23^ Our research findings demonstrated that compared to the NC group, there is an elevated expression of palmitic acid, oleic acid, linoleic acid, and myristic acid in the epilepsy group. Palmitic acid has been shown to play a role in inflammation. A study by Wen et al.^24^ indicated that palmitic acid can activate NOD-like receptor thermal protein domain associated protein 3, which in turn enhances the generation of ROS in macrophages, thereby attenuating the adenosine 5‘-monophosphate-activated protein kinase (AMPK) signal. The AMPK signal serves as a negative regulator of ROS generation and inflammation, thus further increasing ROS production, ultimately resulting in a vicious cycle. Additionally, palmitic acid can also induce ferroptosis in osteoblasts through the activation of the methyltransferase like 3/apoptosis signal-regulating kinase 1-protein 38 signaling pathway.^25^ The relationship between oleic acid and oxidative stress has been extensively studied. Utilizing oleic acid to induce the generation of ROS in HepG2 liver cancer cells is a well-established oxidative stress model.^26^ Furthermore, research has indicated that oleic acid can promote ROS production in mouse dermal fibroblasts, leading to lipid peroxidation.^27^ High concentrations of linoleic acid (500 μg/L) exhibit significant inhibitory effects on algal growth, and studies have shown that the application of high concentrations of linoleic acid to algae results in a significant increase in ROS, potentially promoting ferroptosis.^28^ Moreover, research has demonstrated a positive correlation between the content of myristic acid and ROS levels in tumor cells.^29^ Comparing epilepsy patients to the healthy individuals, there are significant differences in amino acid metabolism as well. Our study reveals a notable decrease in the levels of glutamine and arginine in the epilepsy patients. Looking at the body as a whole, the synthesis of arginine mainly occurs in the epithelial cells of the small intestine. These cells utilize glutamine and glutamate to produce citrulline, which is then converted to arginine with the assistance of renal tubular cells in the kidneys. Therefore, the decrease in arginine can be partly attributed to the decline in glutamine. Glutamine participates in the synthesis of glutathione, enhancing the body’s antioxidant capacity. It serves as an important reducer or antioxidant. Under normal conditions, more than 60% of the free amino acids in the human body appear in the form of glutamine, and the body can produce excess glutamine to meet its needs. However, in many disease states, there is a significant decrease in glutamine, leading to a weakening of the body’s reducing capacity.^30^ We also observed an elevation in bilirubin levels in epilepsy patients. Bilirubin is generated through the breakdown of heme by HO-1, accompanied by the production of carbon monoxide and ferrous iron. Heme iron serves as the primary source of iron in the body, accounting for approximately 67% of total body iron. Therefore, an enhanced metabolism of heme, as indicated by increased bilirubin, naturally leads to changes in iron levels within the body.^15^ Our subsequent *in vivo* experiments in PTZ kindling epileptic mice further demonstrated a significant increase in HO-1 expression and elevated Fe2+ levels in the hippocampal tissue. The upregulation of HO-1 leads to Fe^2+^ overload, intensifies the Fenton reaction, accumulates ROS, and ultimately contributes to the occurrence of ferroptosis.^31^^,^^32^ Studies have reported that FE and TRF are also regulated by HO-1. The upregulation of HO-1 leads to increased levels of ferritin and transferrin, further enhancing iron accumulation.^33^ This finding has been confirmed in our subsequent animal experiments.

Cell death of hippocampal neurons plays a crucial role in the pathogenesis of epilepsy. Currently, research on hippocampal neuron cell death and its association with epilepsy mainly focuses on two aspects: apoptosis and necrotic cell death. In epilepsy, apoptosis may be triggered by abnormal neuronal electrical activity.^34^ Studies have found a significant increase in the extent of neuronal apoptosis during epileptic seizures.^35^^,^^36^ Necrotic cell death is an irregular form of cell death that is often associated with factors such as acute ischemia, hypoxia, and inflammatory responses.^37^ In epilepsy, ischemia and hypoxia are common pathological and physiological alterations, which can contribute to neurodegeneration and necrotic cell death of neurons.^38^ Ferroptosis, initially described by Dixon et al. in 2012, is an iron-dependent regulated cell death pathway that primarily relies on iron-mediated oxidative damage and subsequent membrane disruption, particularly mitochondrial membrane damage.^39^ Ferroptosis of hippocampal neurons plays a crucial role in the pathogenesis of epilepsy; however, the precise molecular mechanisms underlying this process require further investigation.^40^ The essence of ferroptosis is the iron-catalyzed accumulation of lipid ROS, which reacts with membrane polyunsaturated fatty acids, resulting in the accumulation of lipid peroxides and subsequent oxidative cell death.^40^ Ferroptosis differs morphologically and biologically from other types of cell death. Morphologically, during ferroptosis, mitochondria appear smaller with increased membrane density, reduced or vanished cristae, and swelling or even rupture of the outer mitochondrial membrane, while the cell nucleus maintains a normal size without chromatin condensation.^41^ Biologically, ferroptosis is characterized by iron accumulation and redox imbalance resulting from multiple signaling pathways, ultimately leading to lipid peroxidation.^42^ A clinical study conducted by Ikeda et al.^43^ revealed that compared to the healthy control group, patients with epilepsy had significantly higher transferrin saturation in their peripheral blood, suggesting that iron overload in the body may be an important risk factor for epilepsy. Another clinical study in pediatric epilepsy patients found elevated markers of ferroptosis in their peripheral blood, including increased levels of the lipid peroxidation byproduct 4-hydroxy-2-nonenal, partial inactivation of GPX4, and a significant decrease in GSH levels.^44^ Our animal experimental research further supports these findings by demonstrating significant ferroptosis and oxidative stress in hippocampal neurons of the epilepsy animal model, thereby promoting the development of epilepsy. These findings may provide novel targets and strategies for the treatment of epilepsy.

HIF-1, a transcription factor belonging to the HIF-1 family, consists of two subunits, namely HIF-1α and HIF-1β. HIF-1α serves as the primary subunit responsible for functional modulation. HIF-1α exhibits the capacity to regulate the transcriptional activation of more than 60 genes. Its principal role lies in gene expression regulation under hypoxic conditions, facilitating cellular adaptation to oxygen deprivation. The stability and activity of HIF-1α are modulated by various factors, encompassing oxygen levels, intracellular iron content, and activation of other signaling pathways.^11^ In recent years, it has been discovered that in addition to its role in hypoxia adaptation, HIF-1 can also contribute to iron-mediated cell death in certain disease states, such as diabetic nephropathy and malignant mesothelioma.^13^^,^^45^ HO-1, one of the target genes regulated by HIF-1α, is a stress-inducible enzyme. Studies conducted by Feng et al.^13^ have demonstrated that HIF-1α/HO-1-mediated ferroptosis plays a significant role in the pathogenesis of diabetic nephropathy. Furthermore, Wu et al.^14^ have discovered that the environmental pollutant di-2-ethylhexyl phthalate can induce ferroptosis in mouse testes and lead to infertility through the mediation of the HIF-1α/HO-1 signaling pathway. In addition, HIF-1α/HO-1 pathway was also confirmed to promote ferroptosis in the lungs induced by polystyrene nano-plastics.^46^ Hippocampal ferroptosis is a mechanism that triggers neuronal damage and epileptic seizures. However, whether the HIF-1α/HO-1 signaling pathway can mediate hippocampal ferroptosis and contribute to the occurrence and development of epilepsy remains unclear. This study aims to investigate whether the HIF-1α/HO-1 pathway can promote epilepsy by mediating hippocampal ferroptosis. Firstly, the plasma untargeted metabolomic analysis in epileptic patients suggests the occurrence of ferroptosis and activation of the HIF-1α/HO-1 pathway following epileptic seizure. Additionally, analysis of epilepsy disease target genes based on the DisGeNET human disease database further supports HIF-1α as an important disease target gene in human epilepsy. In the subsequent animal experimental validation, our results demonstrate a significant increase in the expression of HIF-1α and HO-1 in PTZ kindling chronic mice elilepsy models. These findings indicate a potential promoting role of HIF-1α/HO-1 in epileptic seizures. Further investigation reveals that the activation of the HIF-1α/HO-1 pathway may positive correlated with abnormal iron metabolism and increased accumulation of Fe^2+^ within hippocampal neurons. Excessive accumulation of Fe^2+^ has been shown to induce oxidative stress and cellular damage, thereby triggering ferroptosis in hippocampal neurons. Furthermore, the activation of the HIF-1α/HO-1 pathway may reduce the activity of antioxidant enzymes in hippocampal tissue, further exacerbating oxidative damage in hippocampal neurons and the progression of epilepsy. In conclusion, our research findings indicated that the HIF-1α/HO-1 pathway might promote the occurrence and development of epilepsy by mediating hippocampal ferroptosis. This discovery provides novel targets and strategies for the treatment of epilepsy. Inhibiting the activation of the HIF-1α/HO-1 pathway and regulating iron metabolism may offer potential approaches for epilepsy treatment. Additionally, enhancing the activity of antioxidant enzymes in hippocampal tissue and boosting the antioxidative ability of hippocampal neurons may have a positive impact on epilepsy therapy.

### Limitations of the study

However, there are several limitations in this study. First of all, the study predominantly establishes correlations between the HIF-1α/HO-1 pathway activation, ferroptosis markers, and epilepsy. However, establishing a causal relationship requires further mechanistic studies, such as targeted genetic or pharmacological interventions to modulate the pathway and observe resultant changes in ferroptosis and epileptic phenotypes. Moreover, more direct and comprehensive brain tissue analysis in human subjects would strengthen the link between the HIF-1α/HO-1 pathway and epileptogenesis. Thirdly, dihydroethidium used in the present study is superoxide-anion specific dye, it cannot able to detect other kinds of ROS, and we intend to include assays that can specifically detect hydrogen peroxide, hydroxyl radicals, and other ROS in the future work.

## STAR★Methods

### Key resources table

**Table:** 

REAGENT or RESOURCE	SOURCE	IDENTIFIER
**Antibodies**		

Rabbit anti-HO-1 monoclonal antibody	Abcam	Cat# AB68477;RRID: AB_11156457
Rabbit polyclonal anti-HIF-1α antibody	Abmart	Cat# P50517R1
Rabbit anti-GPX4 monoclonal antibody	Abmart	Cat# T56959F
Rabbit anti-Transferrin monoclonal antibody	Abmart	Cat# T40111F
Rabbit anti-Ferritin monoclonal antibody	Abmart	Cat# T55648F
Rabbit anti-Cox2 monoclonal antibody	Abmart	Cat# T58852F
Mouse anti-β-actin antibody	Sigma-Aldrich	Cat# A1978; RRID: AB_476692

**Chemicals, peptides, and recombinant proteins**		

PTZ	Sigma-Aldrich	P6500
TranScript One-Step gDNA Removal and cDNA Synthesis SuperMix	TransGen Biotech	AT-311-03
SYBR Premix Ex Taq™ II	Takara	RR820A
Iron Assay Kit	Sigma-Aldrich	MAK025
SOD Assay kit	Beyotime	S0101S
CAT Assay kit	Beyotime	S0051
GPx Assay kit	Beyotime	S0056
Ferritin ELISA Assay Kit	Lai Er Bio-Tech	LE-M2088
Transferrin ELISA Assay Kit	Lai Er Bio-Tech	LE-M1662
DHE	Sigma-Aldrich	D7008
MDA Assay kit	Beyotime	S0131S

**Experimental models: Organisms/strains**		

Male C57BL/6 mice	Yisi Laboratory Animal Technology Co., LTD	NA

**Software and algorithms**		

Prism 8.0	GraphPad	https://www.graphpad.com/
Adobe Illustrator CC 2018	Adobe	https://www.adobe.com/products/illustrator.html
SPSS statistics	IBM	RRID:SCR_002865
Microsoft Excel	Microsoft	RRID:SCR_016137
ZEN 2012	ZEISS	https://www.zeiss.com.cn/
Image J	NIH	https://imagej.net
R version 3.4.1	Vienna	https://www.r-project.org/

### Resource availability

#### Lead contact

Further information and requests for resources and reagents should be directed to and will be fulfilled by the lead contact Songyan Liu (liu_sy@jlu.edu.cn).

#### Materials availability

This study did not generate new unique reagents.

### Experimental model and study participant details

#### Ethics statement

The study was conducted in accordance with the ethical guidelines of the Declaration of Helsinki. The research protocol was approved by the Ethics Committee of China-Japan Union Hospital of Jilin University (Approval Number: 2023022701). Written informed consent was obtained from each patient's next of kin/participant. For animal experiments, the protocols were approved by the Institutional Animal Care and Use Committee of Northeast Normal University in China (Approval Number: AP 20221008).

#### For human studies

Sixty-two epilepsy patients from China-Japan Union Hospital of Jilin University, (Changchun, China) who met the inclusion and exclusion criteria were enrolled in the present study. The entirety of the enrolled participants belonged to the of Han Chinese ethnicity. Furthermore, Table 1 displayed the subjects' age and gender details. The Institutional Review Committee of China-Japan Union Hospital of Jilin University approved the research protocol, The study was conducted in accordance with the ethical guidelines of the Declaration of Helsinki. Written informed consent was obtained from each patient's next of kin/participant.

#### For animal studies

Eight-week-old male C57BL/6 mice were obtained from Yisi Laboratory Animal Technology Co., LTD (Changchun, China). Mice were provided *ad libitum* access to food and water. The mice were housed under a 12-hour light/dark cycle at 25 ± 2°C with a relative humidity of 50 ± 5%.

### Method details

#### Experimental group and plasma samples collection

The inclusion criteria of the present study included: 1. Patients diagnosed with various types of epilepsy according to the 2017 International League against Epilepsy Diagnostic Criteria; 2. Age ≥16 years and follow-up time ≥3 months; 3. Good mental state and ability to cooperate well with examination and treatment; 4. Informed consent of the patient or their guardian. The exclusion criteria as follows: 1. Febrile convulsion, syncope, hysteria and other non-epileptic diseases; 2. Age <16 years, loss of follow-up, or follow-up time <3 months; 3. Serious systemic diseases including liver, kidney, and heart dysfunction, thyroid diseases, and blood system diseases; 4. Alcoholism, family history of mental illness, or inability to cooperate with the examination. Sixty-two epilepsy patients who met the inclusion and exclusion criteria were divided into the infrequent seizures group (IS, n=31) and the frequent seizures group (FS, n=31). Based on our clinical experience, we defined more than three seizures per month as frequent seizures and vice versa as infrequent seizures. Additionally, 20 non-epileptic adults (healthy volunteers) who were recruited during the same period and matched for sex and age were selected as the normal control group (NC, n=20). To avoid the effect of diurnal variation, blood samples were collected in predetermined daytime hours between 9 a.m. and 10:00 a.m. 5 mL of venous blood was collected from all subjects using a heparin anticoagulant tube after fasting on the predetermined time. The collected blood was temporarily stored at 4°C, and within 1 hour, it was sent to the specimen library of the China-Japan Union Hospital of Jilin University for plasma preparation and frozen at −80°C.

#### Plasma untargeted metabolomics analysis

##### Plasma samples preparation for metabolomics

The plasma samples were prepared by thoroughly mixing 100 μL of the sample with 400 μL of cold methanol acetonitrile (v/v, 1:1) using vortexing. The mixture was sonicated for 1 h in ice baths, incubated at -20°C for 1 h, and centrifuged at 4°C for 20 min at a speed of 14,000 g. The resulting supernatants were harvested and dried under vacuum for UHPLC-MS/MS analysis.

##### UHPLC-MS/MS analysis

Metabolomics profiling was performed using a UPLC-ESI-Q-Orbitrap-MS system consisting of a UHPLC (Shimadzu Nexera X2 LC-30AD, Shimadzu, Japan) coupled with a Q-Exactive Plus mass spectrometer (Thermo Scientific, San Jose, USA). For hydrophilic interaction liquid chromatography separation, the samples were analyzed using a 2.1 mm × 100 mm ACQUIY UPLC BEH Amide 1.7 μm column (Waters, Ireland). The flow rate was set at 0.5 mL/min, and the mobile phase consisted of two components: A) 25 mM ammonium acetate and 25 mM ammonium hydroxide in water, and B) 100% acetonitrile (ACN). The gradient elution started at 95% B for 1 min, followed by a linear decrease to 65% B over 7 min, then to 35% B in 2 min and maintained for 1 min, and finally increased to 95% B in 0.5 min, with a 2 min re-equilibration period. Both electrospray ionization (ESI) in positive ion mode and negative ion mode were used for MS data acquisition. The HESI source conditions were set as follows: Spray Voltage: 3.8 kV (+) and 3.2 kV (-); Capillary Temperature: 320 (±); Sheath Gas: 30 (±); Aux Gas: 5 (±); Probe Heater Temp: 350 (±); S-Lens RF Level: 50. In MS-only acquisition mode, the instrument was set to acquire data in the m/z range of 80-1200 Da. Full MS scans were performed at a resolution of 70,000 at m/z 200, and MS/MS scans were performed at a resolution of 17,500 at m/z 200. The maximum injection time was set to 100 ms for MS and 50 ms for MS/MS. The MS2 isolation window was set to 2 m/z, and the normalized collision energy (stepped) was set to 27, 29, and 32 for fragmentation. Quality control (QC) samples were prepared by pooling representative aliquots from all samples and used for data normalization. Blank samples (75% ACN in water) and QC samples were injected every sixth sample during acquisition.

##### Data preprocessing and filtering

The raw MS data from the NC, IS, and FS groups were processed using MS-DIAL for peak alignment, retention time correction, and peak area extraction. Metabolites were identified based on accurate mass (mass tolerance < 0.01 Da) and MS/MS data (mass tolerance < 0.02 Da), which were matched against the Human Metabolome Database (HMDB), MassBank, and other public databases, as well as a self-built metabolite standard library from Bioprofile Biotechnology (Shanghai, China). Only variables with more than 50% non-zero measurement values in at least one group were retained in the extracted-ion features.

##### Multivariate statistical analysis

We utilized R version 3.4.1 (R Foundation for Statistical Computing, Vienna, Austria) for all multivariate data analyses and modeling. The data were mean-centered using Pareto scaling. We constructed models using Orthogonal Partial Least Squares Discriminant Analysis (OPLS-DA). Consequently, a score plot was generated to observe the separation among the NC, IS, and FS groups. The variable importance in projection (VIP) scores established by OPLS-DA were utilized to select the metabolites that exhibited the highest discriminatory power within each group. A VIP score greater than 1 was defined as the threshold for the current analysis. We conducted a one-way ANOVA test analysis to compare the abundance of metabolites among the three groups. A significance level of P-value < 0.05 was applied in the OPLS-DA analysis to identify differentially abundant metabolites. Subsequent to that, we generated HMDB classification ring maps using these differentially abundant metabolites as the basis. Additionally, we employed Welch's t-test analysis to compare the abundance of metabolites between the two groups.

##### Kyoto Encyclopedia of Genes and Genomes (KEGG) enrichment analysis

To identify disrupted biological pathways, we conducted KEGG pathway analysis using the KEGG database (http://www.kegg.jp) with the differential metabolite data from the NC, IS, and FS groups. KEGG enrichment analyses were conducted using the Fisher's exact test, and multiple testing correction was applied using the FDR method. The enriched KEGG pathways showed nominal statistical significance at a p-value < 0.05 threshold.

#### Screening and functional enrichment analysis of epilepsy-associated genes based on DisGeNET database

1. Retrieve epilepsy-associated gene targets

Enter "epilepsy" in the DisGeNET database (https://www.disgenet.org/) and download the summary files of gene-disease associations.

2. Protein-Protein Interaction (PPI) network analysis

Epilepsy-associated target genes obtained from the DisGeNET database were imported into the String database (https://cn.string-db.org/). The network was constructed by selecting "Multiple proteins" and "Homo sapiens," resulting in a network diagram consisting of 1122 nodes and 22654 edges. Utilizing the MCODE plugin (Cytoscape 3.7.2 plugin), the following settings were applied: "In whole network," "degree cutoff = 2," "Haircut" with "node score cutoff = 0.2, K-core = 2, Max. Depth = 100." Finally, the gene targets ranked first in terms of "K-core" were selected as the hub targets for epilepsy.

3. Functional enrichment and pathway analysis

The key target genes of epilepsy were initially uploaded into the online software, David (https://david.ncifcrf.gov/), with the settings "H. sapiens" and "P < 0.05." Subsequently, Gene Ontology (GO) enrichment analysis was performed by selecting GO Biological Processes (BP), GO Molecular Functions (MF), and GO Cellular Components (CC). The GO enrichment results were visualized as a histogram based on the P-value (TOP30) for analysis. In addition, KEGG enrichment analysis was performed by selecting KEGG Pathway. The enrichment results of KEGG pathways were visualized as a bubble map based on the P-value (TOP30) for analysis.

#### Establishments of pentylenetetrazol (PTZ) kindling epilepsy models

The mice were divided into two groups: (1) PTZ group: C57BL/6 mice were intraperitoneally injected with PTZ (P6500, Sigma-Aldrich, USA) at a dose of 35 mg/kg once every other day for a total of eleven injections.^47^ Mice showing more than three consecutive stage 4 seizures were considered kindled. (2) Control group: Control C57BL/6 mice were simultaneously injected with saline. Seizure behavior was video-monitored for 30 minutes after each injection. Seizure intensity was assessed using the modified Racing scale as follows: Stage 0, no response; Stage 1, ear and facial twitching; Stage 2, convulsive twitching axially through the body; Stage 3, myoclonic jerks and rearing; Stage 4, wild running and jumping; Stage 5, generalized tonic-clonic seizures; and Stage 6, death.^48^^,^^49^

#### Hematoxylin-eosin (HE) staining

For HE staining, mice were perfused with cold PBS followed by 4% paraformaldehyde (PFA) through transcardial perfusion. The mice brains were post-fixed overnight at 4°C and subsequently transferred to a 30% sucrose solution. Next, the brains were embedded in paraffin. Subsequently, tissue blocks were sectioned into 6 μm slices and stained with hematoxylin and eosin. Representative images were captured using an Olympus FSX100 microscope.

#### Transmission electron microscope (TEM)

For TEM assay, mice were transcardially perfused with heparinized normal saline, followed by perfusion with a mixture of 2.5% glutaraldehyde and 1% PFA in 0.1 M phosphate buffer (PB, pH 7.4). Next,the hippocampus was dissected and subjected to TEM analysis.^50^ Briefly, the hippocampus was dissected from the entire brain. Next, the dissected hippocampal tissue samples were diced and fixed in 2.5% glutaraldehyde. Then, the hippocampus was transversely sectioned at 70 μm using a vibratome. The sections were then treated with 0.5% OsO4 (in 0.1 M PB) for 1 hour, dehydrated in a series of graded alcohols, flat-embedded in EMbed 812 (Electron Microscopy Sciences, Hatfield, PA), and cured for 48 hours at 60°C. Ultrathin sections of 60 nm were cut and mounted on Formvar-coated single-slot grids. The sections were stained with uranyl acetate and lead citrate, and examined under an electron microscope (HT-7800, Hitachi, Tokyo, Japan). As for quantification, the Image-Pro Plus 6.0 software was used to select 5 mitochondria under each field of view and measure their length (μm) and area (μm^2^) with a 2.5k 5μm scale as the standard. 5 fields of view are selected for each sample.

#### Real-time quantitative PCR (RT-qPCR)

RT-qPCR assays were performed as a previously described protocol.^51^^,^^52^ Specifically, total RNA was extracted from mouse hippocampus using TRIzol reagent (257401, Invitrogen, USA). Complementary DNA (cDNA) was synthesized using the TranScript One-Step gDNA Removal and cDNA Synthesis SuperMix (AT-311-03, TransGen Biotech, Beijing, China) according to the manufacturer’s protocol. RT-qPCR was performed using SYBR Premix Ex Taq™ II (RR820A, Takara) using the ABI StepOnePlus Real-Time PCR System. Each sample was analyzed in triplicate, and the mRNA levels were normalized to the β-actin mRNA level using the 2^−ΔΔCT^ method. Melting curves were automatically recorded and analyzed for each reaction. The primer sequences are listed in the below table.

**Table:** Primer sequences information

Gene	Primer sequences
*β-actin* -F	5′- GGT GAA GGT CGG TGT GAA CG -3′
Mouse*-β-actin* -R	5′- CTC GCT CCT GGA AGA TGG TG -3′
Mouse*-HIF-1α*-F	5′- GAATGAAGTGCACCCTAACAAG -3′
Mouse*-HIF-1α*-R	5′- GAGGAATGGGTTCACAAATCAG -3′
Mouse*-HO-1* -F	5′- CCGCTACCTGGGTGACCTCTC -3′
Mouse*-HO-1* -R	5′- GACGAAGTGACGCCATCTGTGAG -3′
Mouse*-GPX4*-F	5′- CATGCCCGATATGCTGAGTGTGG -3′
Mouse*-GPX4*-R	5′- TAGCACGGCAGGTCCTTCTCTATC -3′
Mouse*-FTH1*-F	5′- TGCCATCAACCGCCAGATCAAC -3′
Mouse*-FTH1*-R	5′- ATTCAGCCCGCTCTCCCAGTC -3′
Mouse*-Transferrin* -F	5′- GGA CGC CAT GAC TTT GGA TG -3′
Mouse*-Transferrin* -R	5′- GCC ATG ACA GGC ACT AGA CC -3′
Mouse*-PTGS2* -F	5′- TTCCAATCCATGTCAAAACCGT -3′
Mouse*-PTGS2* -R	5′- AGTCCGGGTACAGTCACACTT -3′

#### Protein extraction and western blotting

Mouse hippocampal tissues were subjected to protein extraction using a modified RIPA buffer (50 mM Tris-HCl, pH 7.4, 150 mM sodium chloride, 1% NP-40, 0.25% sodium deoxycholate, and proteinase inhibitors). Protein concentrations were measured using a BCA Protein Assay Kit (P0012, Beyotime, Shanghai, China). Western blotting was conducted following a previously described protocol.^51^ Specifically, the protein samples were separated by SDS-PAGE and subsequently transferred onto PVDF membranes (Millipore). The membranes were then incubated overnight at 4°C with a specific primary antibody, followed by incubation with the corresponding HRP-conjugated secondary antibody. The chemiluminescent signals were visualized using ECL Prime Western Blot Detection reagent (GE Healthcare). The ECL signal was detected using a Tanon-5200 system (Tiangen (Beijing) Biotech Co., Ltd.) at the appropriate exposure time points. Band intensities were quantified using ImageJ software (NIH), and the values were normalized to the expression level of β-actin in each sample. The following antibodies were used in the study: Rabbit anti-Heme Oxygenase 1 monoclonal antibody (Abcam, AB68477, 1:10000); Rabbit polyclonal anti-HIF-1α antibody (Abmart, P50517R1, 1:1000); Rabbit anti-GPX4 monoclonal antibody (Abmart, T56959F, 1:1000); Rabbit anti-Transferrin monoclonal antibody (Abmart, T40111F, 1:1000); Rabbit anti-Ferritin monoclonal antibody (Abmart, T55648F, 1:1000); Rabbit anti-Cox2 monoclonal antibody (Abmart, T58852F, 1:1000); Mouse anti-β-actin antibody (Sigma-Aldrich, A1978, 1:5000); HRP-conjugated secondary antibodies against mouse or rabbit were obtained from Invitrogen (Carlsbad, USA).

#### ELISA

The levels of Ferritin and Transferrin^2^ proteins in mouse hippocampus lysates were quantified using ELISA Assay Kits (LE-M2088 and LE-M1662, Lai Er Bio-Tech, Anhui, China) following the manufacturer's instructions. The OD values were measured at 450 nm using a Milton Roy Spectronic 3000 Array spectrophotometer. The total protein concentration in each sample was determined using the Bradford method.

#### Ferrous iron (Fe^2+^) assay

The relative concentration of Fe^2+^ in the mouse hippocampus tissue lysates was determined using an Iron Assay Kit (MAK025, Sigma-Aldrich, USA) following the manufacturer's instructions. OD values were measured at 593 nm using a Milton Roy Spectronic 3000 Array spectrophotometer. T The total protein content in each sample was determined using the Bradford method.

#### Antioxidant enzyme activity assay

The relative activity of Superoxide Dismutase (SOD), catalase (CAT), and Glutathione Peroxidase (GPx) in the mouse hippocampus tissues was examined using commercial test kits (S0101S, S0051, and S0056, Beyotime, Shanghai, China) following the manufacturer's protocols. The absorbance was measured at 450 nm for SOD activity, 340 nm for GPx activity, and 520 nm for CAT activity using a Milton Roy Spectronic 3000 Array spectrophotometer. The total protein content in each sample was measured using the Bradford method.

#### Reactive oxygen species (ROS) staining

For ROS staining, mice were sacrificed, and the whole brain was immediately dissected, snap-frozen, and stored at -80°C. The brain samples were embedded in optimal cutting temperature compound (G6059, Wuhan servicebio technology CO., LTD, China) and frozen. Frozen sections of 8 μm thickness were prepared using a cryostat microtome (CRYOSTAR NX50; Thermo Fisher Technology (China) Co., LTD). Then, the brain slices were washed with distilled water, and were incubated in dihydroethidium (DHE) staining solution (D7008, Sigma-Aldrich, USA) for 30 min. Subsequently, the slices were immersed in nucleus staining solution (G1012, Wuhan servicebio technology CO., LTD, China) for 10 min, followed by washing with PBS (pH 7.4). Finally, the slices were used for measuring ROS content after being washed with PBS (pH 7.4) again. The ROS-stained sections were observed using a Zeiss LSM 780 confocal microscope with ZEN 2012 software. After imaging, only the brightness, contrast, and color balance were adjusted. The ROS fluorescence intensity in different regions of the hippocampus was measured using ImageJ software (Image J, NIH, Bethesda, MD, USA).

#### Lipid peroxidation assay

The relative concentration of malondialdehyde (MDA) in the mouse hippocampus lysates was determined using a Lipid Peroxidation MDA Assay Kit (S0131S, Beyotime, Shanghai, China) following the manufacturer's instructions. OD values were measured at 532 nm using a Milton Roy Spectronic 3000 Array spectrophotometer. The total protein content in each sample was measured using the Bradford method.

### Quantification and statistical analysis

The statistical analysis was performed using SPSS 22.0 software. After confirming normal distribution, unpaired two-tailed Student's t-test was used to compare two groups (P > 0.05). One-way ANOVA was used to compare three or more groups, followed by conducting pairwise comparisons between groups using Welch’s T-test. The data are presented as mean ± standard error of the mean (SEM). P values ≤ 0.05 were considered statistically significant. Graphs were generated using GraphPad Prism 8 for Windows or Microsoft Excel for Windows. ∗P<0.05, ∗∗P<0.01, ∗∗∗P<0.001, and ns for no significance.

## Data Availability

Data reported in this paper will be shared by the lead contact upon request. This paper does not report original code. Any additional information required to reanalyze the data reported in this paper is available from the lead contact upon request.

## Supplemental information

Table S1. Significant Differential Metabolites in positive and negative mode, related to Figures 1 and 2

Table S2. Epileptic target genes in the DisGeNET database, related to Figure 3A

Table S3. GO enrichment analysis results, related to Figure 3B

Table S4. KEGG enrichment analysis results, related to Figure 3C
